# Anti-Aging Effect of Chitosan Oligosaccharide on d-Galactose-Induced Subacute Aging in Mice

**DOI:** 10.3390/md16060181

**Published:** 2018-05-24

**Authors:** Song-Zhi Kong, Ji-Cheng Li, Si-Dong Li, Ming-Neng Liao, Cheng-Peng Li, Pin-Jin Zheng, Min-Hui Guo, Wei-Xiang Tan, Zhao-Hui Zheng, Zhang Hu

**Affiliations:** Faculty of Chemistry and Environmental Science, Guangdong Ocean University, Zhanjiang 524088, China; kongsongzhi@126.com (S.-Z.K.); 15702098206@163.com (J.-C.L.); 13702737491@163.com (S.-D.L.); gdoulmn@163.com (M.-N.L.); lcp0802@126.com (C.-P.L.); aa904855159@163.com (P.-J.Z.); guominhui18@126.com (M.-H.G.); waysangtam@sina.com (W.-X.T.); zhengzh1017@163.com (Z.-H.Z.)

**Keywords:** chitosan oligosaccharide, d-galactose, subacute aging, anti-aging, anti-oxidant, immune function

## Abstract

Chitosan oligosaccharide (COS), a natural polysaccharide with good antioxidant and anti-inflammatory properties, is the depolymerized product of chitosan possessing various biological activities. The present study was designed to investigate the possible anti-aging effect of COS on the aging model mouse induced by d-galactose (d-gal) and explore the underlying mechanism. In the experiment, 48 male Kunming mice (KM mice) were randomly divided into the normal group, model group, positive group, and low-medium-high dose polysaccharide groups (300, 600, 1200 mg/kg/day). The results showed that COS, by intragastric gavage after subcutaneous injection of d-gal (250 mg/kg/day) into the neck of mice consecutively for eight weeks, gradually recovered the body weight, the activity of daily living, and organ indices of mice, as well as effectively ameliorated the histological deterioration of the liver and kidney in mice triggered by d-gal. To be specific, COS obviously improved the activities of antioxidant enzymes in liver and kidney of KM mice, including catalase (CAT), glutathione peroxidase (GSH-Px), and superoxide dismutase (SOD), as well as decreased malondialdehyde (MDA) levels when compared with those in model group mice. Furthermore, COS not only elevated the diminished levels of serum immunoglobulin G (IgG) and IgM induced by d-gal, but also significantly inhibited the d-gal-caused upregulation of serum alanine aminotransferase (ALT), aspartate transaminase (AST), alkaline phosphatase (ALP), uric acid (UA) and creatinine (CREA) levels as compared with those of mice in the model group. These results demonstrate that COS has an obvious anti-aging activity in d-gal-induced subacute aging mice, the mechanism of which, to some extent, is associated with enhancing the antioxidant defenses, reducing oxidative stress, and improving the immune function of aging model mice.

## 1. Introduction

Aging, also known as senescence, is a complicated and irreversible degeneration that appears not only in body tissues, but also in the physiological functions of body organs, such as poor immune responses, memory loss, and so on. Numerous studies have discovered that aging is closely associated with diverse chronic diseases, such as hypertension, diabetes, atherosclerosis, Alzheimer’s disease, Parkinson’s disease, and multiple cancers [1,2,3]. After entering the 21st century, with the trend of global aging and the improvement of living standards all over the world, health problems caused by aging and aging-associated diseases have become increasingly prominent. Therefore, how to maintain the health of the elderly and delay the occurrence of aging and aging-related diseases has already become a common topic among people today, especially among the young, as well as a research hotspot for scientists [2,3]. In the theoretical hypotheses of aging, the free radical theory of aging, proposed initially by Denham Harman since the 1950s, has been widely concerned [4]. It is well-accepted that the most significant determinant of aging is oxidative damage caused by overproduction of reactive oxygen species (ROS). The formation of excess ROS directly results in oxidative stress and pro-inflammatory responses, which then causes redox disequilibrium, ultimately leading to protein oxidation, lipid peroxidation, and mitochondria and DNA damage, which can precisely inhibit normal cell functions, and even cause apoptosis [5,6]. Meanwhile, ROS is expected to play an additional role in aging by directly or indirectly damaging the endogenous antioxidant system, including superoxide dismutases (SOD), catalase (CAT), and glutathione peroxidase (GSH-Px) [7]. In addition, more and more evidence has demonstrated that aging is an inevitable biological phenomena caused by the decline of body immune function, and studies have also shown that there is a positive relationship between aging and immune dysfunction, which leads to a decrease in the ability of the body to resist external pathogens, thereby increasing the possibility of aging-related diseases [8,9,10]. Based on the analysis of these aging mechanisms, the agents with anti-oxidant and/or immunomodulatory functions might be of preventive or potential therapeutic value in aging-associated diseases.

**Figure 1:**
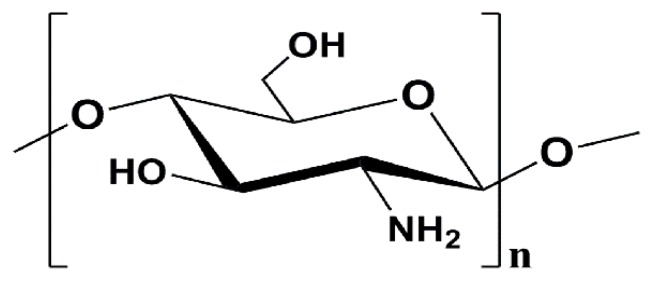
Chemical structure of COS.

Recently, numerous natural compounds have been discovered to possess potent anti-aging activity and have attracted considerable interest as potential candidates for the development of novel anti-aging therapies [6,7,9]. Consequently, close attention has been paid to natural products due to their amicable safety profile and potentially beneficial biological activities, thus, searching natural ingredients with anti-oxidant, immunomodulatory and anti-inflammatory properties for preventing and/or treating this subacute aging has become a new hot spot. Chitosan oligosaccharide (COS), consisting of 2–10 β-1,4-linked d-glucosamine units, is the depolymerized product of chitosan (as displayed in Figure 1) [11]. Studies have displayed that COS is biocompatible, non-toxic, and absorbable, as well as has a variety of biological effects, including anti-bacterial, neuroprotective, anti-diabetic, anti-tumoral and anti-hypoglycemic properties, which grant it potential in pharmaceutical applications [11,12,13]. Furthermore, there is also evidence showing that COS can enhance antioxidant activity, maintain a favorable redox balance, and possess anti-inflammatory capacity by adjusting relevant signaling pathways [14,15]. Our recent study has demonstrated that the ultraviolet irradiation-induced photoaging on hairless mouse skin is significantly ameliorated by topical treatment with COS [16]. Additionally, it has been reported that COS can attenuate oxidative stress-induced retinal damage in rats, protect mice from LPS challenge, prevent retinal I/R injury in rats and accelerate weaned pig growth mainly by maintaining the activities of antioxidative enzymes and inhibiting the activation of NF-κB [17,18,19,20]. In addition, it is widely acknowledged that, with low molecular weight and low viscosity, COS has a great influence on the absorption of the human intestinal tract, and can quickly enter the bloostream; meanwhile, it also contributes to producing a systemic biological effect in the organism and enhance the pharmacological effects in the body [17,18]. 

Based on the previous studies on the biological activities of COS, it is reasonable to believe that this oligosaccharide may possess a considerable potential to be utilized in the anti-aging field. However, there is limited knowledge about the protective effects of this natural polysaccharide with low molecular weight against d-galactose (d-gal)-induced subacute aging in vivo. Therefore, the present study aimed to investigate the anti-aging activity of COS by intragastric gavage in d-gal-induced subacute aging mice mainly via measuring the oxidative stress parameters, as well as evaluating the macroscopic and histopathological statuses of mice and/or their main organs to provide a pioneering pharmacological basis for the anti-aging effect of COS. 

## 2. Results

### 2.1. Daily Behavior Observation

**Figure 2:**
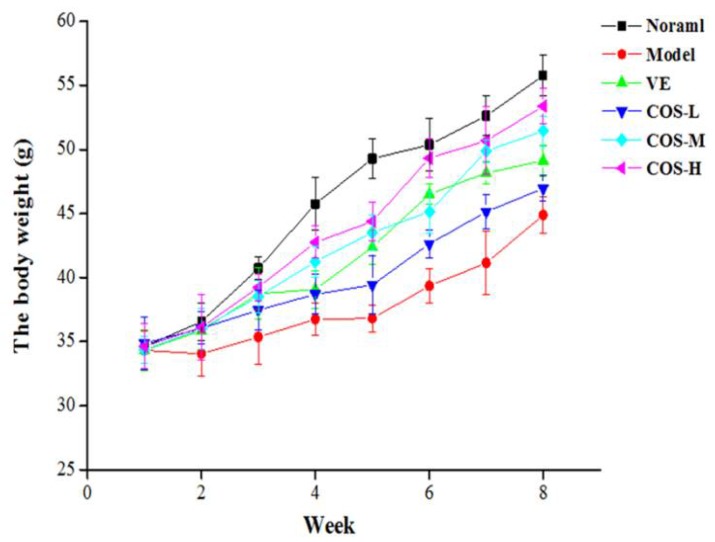
The body weight of mice during the treatment period.

During the eight-week period of our experiment, the daily appearance and behavioral activities of the mice were observed and recorded weekly, which showed that there were no pathological changes, such as vomiting and anti-feeding, occurring in each group. Mice in the normal group were not only in a good state of mind, but also lively, active, and agile, as well as had a healthy diet. As exhibited in Figure 2, except for the normal group, mice in other groups showed weight loss at the initial stage after d-gal injection, and then gradually recovered weight. After the fifth week of d-gal injection, mice in the model group showed evident symptoms of aging, including slow movement, gradually decreased food intake, a lag in response, listlessness, as well as withered and lackluster fur, indicating that the aging model triggered by subcutaneous d-gal injection was successfully established. Compared with the model group, these above senescence characteristics of mice in vitamin E (VE) and drug administered groups were receded to differential extents. Especially in the COS-H treatment group, the aging features of mice hereinabove were significantly moderated as compared to those of model group mice. Although there was slightly lackluster fur and a slow reaction for mice in low- and middle-dose COS groups, the phenomenon of senescence was obviously moderated in comparison with that in the model group. 

### 2.2. Effect of COS on Immune Function

Since the spleen and thymus are prominent immune organs, the indices of them can reflect the strength of the immune status or the improvement of immune function [10]. As shown in Table 1, after an eight-week treatment of d-gal, the spleen and thymus indices of the model group mice were noticeably lower than those in the normal group, indicating that the mouse immune system was inhibited by the administration of d-gal for 56 consecutive days. Moreover, the decreased spleen and thymus indices of mice induced by d-gal treatment were gradually up-regulated to a certain degree after COS administration. Furthermore, our results (as revealed in Table 1) also revealed that the serum levels of immunoglobulin G (IgG) and IgM visibly decreased in model group mice when compared to those in the normal group. Nevertheless, after being gavaged with COS, the levels of IgG and IgM were clearly elevated in a dose-dependent manner.

### 2.3. Effect of COS on Liver and Kidney Function

The effect of COS on liver function in d-gal-treated mice was assessed by measuring the serum levels of alanine aminotransferase (ALT), aspartate transaminase (AST), and alkaline phosphatase (ALP). As seen in Table 2, after treated with d-gal for eight weeks, mice in the model group exhibited marked increases in the serum levels of ALT, AST, and ALP when compared with normal mice (all *p* < 0.05). However, these d-gal-triggered unusually high levels of ALT, AST, and ALP in mice serum were gradually decreased to a certain extent in VE and COS-treated groups when compared to model group mice, especially for the COS-H group, and these levels were markedly lower than those in the model group (all *p* < 0.05).

Meanwhile, the effect of COS on kidney function in d-gal-injected mice was evaluated by measuring the levels of creatinine (CREA) and uric acid (UA) in their serum, and the results are also exhibited in Table 2, which reveals that the serum levels of CREA and UA for the model group mice are significantly higher than the normal group mice (all *p* < 0.05). Nevertheless, for the VE and COS-administrated groups, these unfavorable increases of serum CREA and UA levels are gradually restored to an extent lower than those of the model group (especially in the COS-H group).

### 2.4. Effect of COS on d-Gal-Induced Histochemical Damages of the Liver and Kidney

**Figure 3:**
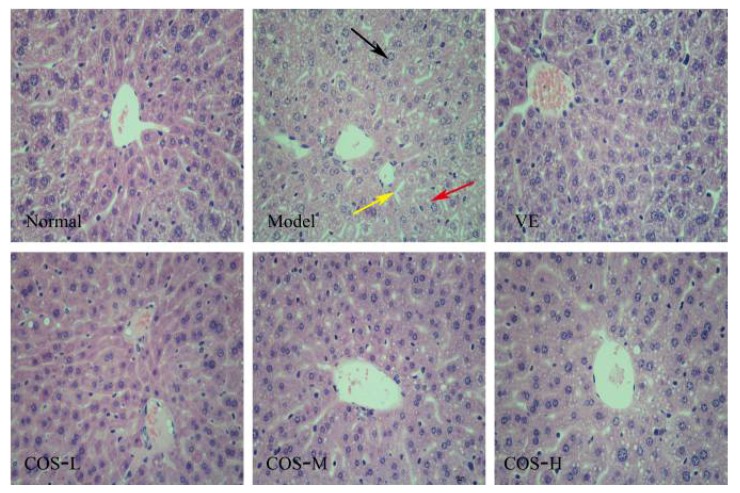
Histopathological appearance of liver (haematoxylin-eosin (HE) staining 400×). Binucleation of hepatocyte (black arrow), hepatic cord arranged mussily (yellow arrow), and the ballooning hepatocyte (red arrow).

As shown in Figure 3, the normal histological structure of liver tissue is clearly visible in the normal group mice, including ubiquitous polygonal hepatocytes, radially-ranged hepatic cord around a central vein and slight binucleation of hepatocytes. After eight-week continuous d-gal-injection, the liver tissue revealed an increased number of binucleation of hepatocytes, mussily-arranged hepatic cord, widespread hepatocellular ballooning, and a number of inflammatory cell infiltrations. Nevertheless, for the COS-H-treated group, neatly arranged hepatic cord, fewer inflammatory infiltrations and binucleation of hepatocytes were observed in mice liver tissues, while the ballooning hepatocytes were decreased in a dose-dependent manner, indicating that COS could improve d-gal-induced liver injury to some extent. 

**Figure 4:**
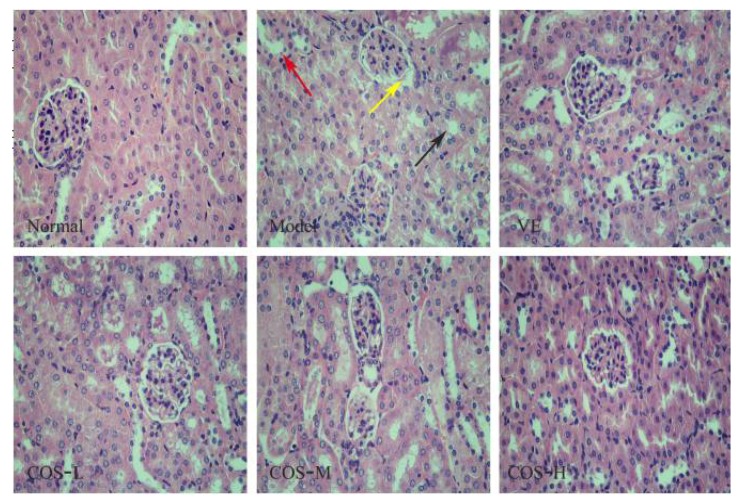
Histopathological appearance of kidney (HE staining 400×). Atrophy (yellow arrow), drop of epithelial cells in renal proximal convoluted tubules (black arrow), and balloon widened cavity (red arrow).

In addition, histopathological analyses of kidney tissue of d-gal-treated mice revealed that the glomerulus showed obvious atrophy, or even disappeared, while the balloon-widened cavity and drop of epithelial cells in renal proximal convoluted tubules, as well as extensive renal edema, could occur when compared to the normal group (as revealed in Figure 4). Notably, COS and VE treatment significantly attenuated these unhealthy pathological changes of kidney tissue caused by d-gal, manifesting kidney-protecting activity, while renal edema and the balloon-widened cavity were largely controlled for the COS-H group. These results showed that COS successfully alleviated these d-gal-induced pathological damages in liver and kidney tissues of Kunming mice (KM mice).

### 2.5. Effect of COS on Anti-Oxidation Indices of Liver and Kidney

**Figure 5:**
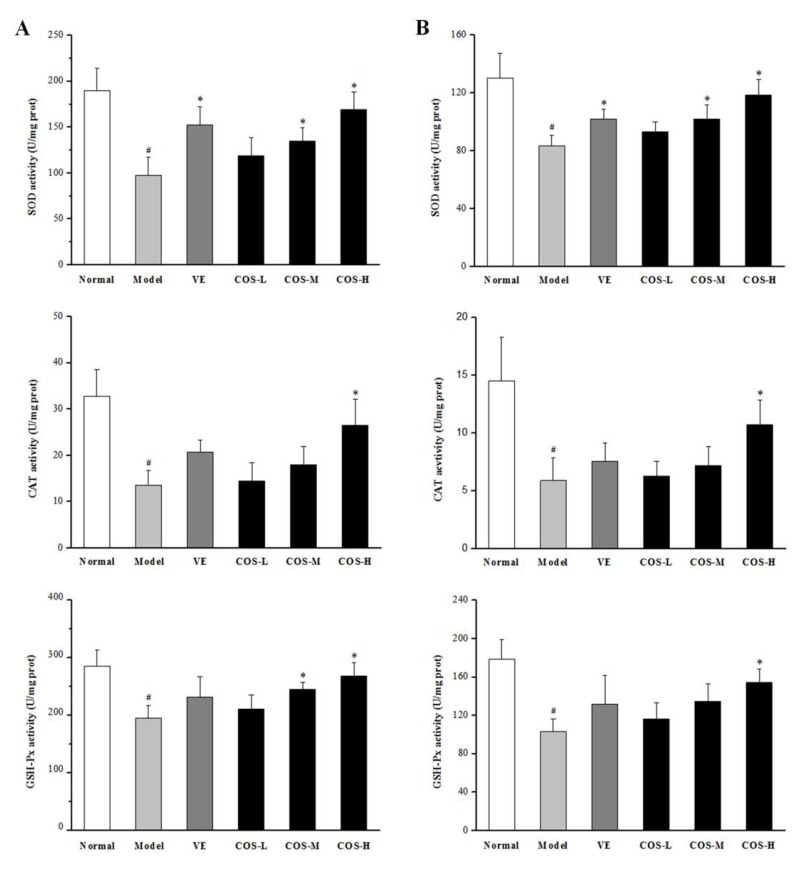

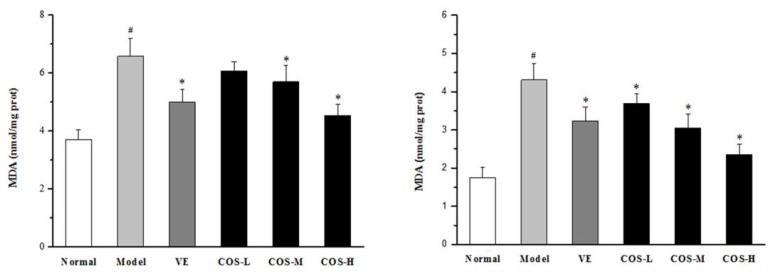
Effect of COS on the activities of SOD, GSH-Px, and CAT, as well as MDA levels of mice liver (**A**) and kidney (**B**) caused by d-gal. Data represent means ± SD (n = 8). **^#^** *p* < 0.05 compared with the normal group; * *p* < 0.05 compared with the model group. Normal: normal control group; Model: d-gal-treated group; VE: d-gal plus VE treatment group; COS-L: d-gal plus COS (300 mg/kg/day) treatment group; COS-M: d-gal plus COS (600 mg/kg) treatment group; COS-H: d-gal plus COS (1200 mg/kg) treatment group.

Previous studies have shown that antioxidative enzymes can protect the liver and kidney from d-gal-induced subacute aging by scavenging ROS. Hence, the activities of SOD, CAT, and GSH-Px, as well as the content of malondialdehyde (MDA), were investigated in our present study [5,6,7]. As illustrated in Figure 5, after subcutaneous injection of d-gal for eight weeks, the activities of these three key antioxidative enzymes were sharply decreased in comparison with those in liver and kidney tissues of normal group mice, while the opposite was true for the MDA level (all *p* < 0.05). However, when the mice were administrated with COS, the decreased activities of these enzymes caused by d-gal were gradually elevated to an extent higher than those of the model group mice, and the MDA level was attenuated by COS in a dose-dependent manner (especially in COS-H group, *p* < 0.05 vs. model group). These results suggested that COS treatment could significantly improve SOD, CAT, and GSH-Px activities and decrease the MDA level in the liver and kidney of d-gal-treated mice. 

## 3. Discussion

It has been widely accepted that the d-gal-induced subacute aging mouse model used in our present study is a usual adopted aging model based on the metabolic theory of aging, the symptoms of which are similar to the natural aging process. d-gal is a normal nutrient that naturally exists in the body [21]. As a certain dose of d-gal is injected into the mice within a period of time, the concentration of it in cells will be too high to be catalyzed by galactose oxidase to aldose and hydrogen peroxide, finally generating superoxide anions [22]. The oxidation in the body produces a large number of free radicals, which are beyond the body’s scavenging capacity and lead to lipid peroxidation; meanwhile, the final decomposition products (such as MDA) can directly or indirectly combine with proteins, nucleic acids, phospholipids, and other substances, not only destroying the chemical structure of intracellular life substances and disrupting cell function, but also damaging normal tissue cells, as well as affecting the normal osmotic pressure, which further lead to metabolic disorders of vital organs and eventually organism aging [23,24]. Thus, d-gal-induced subacute aging in mice has been chosen in our current study to investigate the possible anti-aging effects of COS and explore the underlying mechanism. In the present study, the results revealed that the model group mice had a significant difference in their daily behaviors compared with those of normal group mice. In addition, the spleen and thymus of model mice significantly atrophied, while the indices of their spleen and thymus were significantly lower than those of normal mice. Moreover, vitalities of antioxidant enzymes, such as GSH-Px, CAT, and SOD in the liver and kidney of model mice were reduced, while the opposite was true for the MDA level. These results are in line with previous studies [6,7,22,23,24], indicating that the d-gal-induced aging model in mice was successfully established in our present study. 

With the increase of age, immune system of the body suffers degenerative changes that not only decreases the immune response to a foreign specific antigen, but also presents with a general imbalance of immune function, which finally led to the occurrence of various diseases [25]. Therefore, the regulation of body immunity is one of the main methods in the anti-aging study. Additionally, the thymus and spleen are two important immune organs of the body, the organ indices of which can initially reflect the strength of a non-specific immune system, and which are also the preliminary indices to estimate the non-specific immune function of the body [26,27]. The present results showed that compared with the normal group, the immune organs, such as the thymus and spleen of mice in the model group injected with d-gal for eight weeks, were obviously shrunk, and the thymus and spleen indices were distinctly decreased. When compared with the model group, although, for VE-treatment, there was no obvious promotion impact to these d-gal-induced diminution of thymus and spleen indices, these two organs’ indices in the COS low-middle-high dose groups gradually increased (especially for the spleen index), indicating that COS could improve the spleen and thymus quality indices of senile mice, inhibit the degeneration of the immune organs, and ultimately enhance the non-specific immunity to some degree. On the other hand, as one of the nonspecific immune factors of the body, the antibody system plays an important role in the body’s immune response and immune adaptation, while immunoglobulin (Ig) is a commonly used indicator of humoral immune status [28,29,30]. Serum Ig includes IgG, IgM, IgA, IgD, and IgE, of which IgG and IgM are the main components of serum antibody, as their content can reflect immune response capacity and humoral immunity of the body [31]. The results in the present study showed that the serum levels of IgG and IgM in the model group mice were significantly lower than those in normal group mice. Compared with those of the model group, for VE-treatment, there was also no marked promoting effect on these d-gal-produced reduction of IgG and IgM levels in mice serum, while these serum levels of IgG and IgM of senescent mice treated with COS increased significantly, indicating that COS could improve the immune response ability and enhance humoral immune function of the body. In summary, COS has a good regulatory role on immune function of d-gal-induced aging mice, which supposedly serves to delay aging.

In addition, the endogenous antioxidant enzyme system, mainly including SOD (an enzyme devoted to scavenge superoxide anion radicals and reduce the production of lipid peroxidation), CAT (an enzyme dedicated to scavenge H_2_O_2_ to protect peroxidation of cell wall lipid and lipoproteins), and GSH-Px (an enzyme concentrated on catalyzing GSH into GSSG and stimulating the toxic peroxide reduction into non-toxic hydroxyl compounds), is an important defense system against free radical damage in the body, the main function of which is to maintain the homeostasis of ROS in the internal environment and to remove excessively high levels of ROS [4,5,32,33]. Numerous evidences exhibit that the excessive production of ROS in biological systems can cause oxidative damage to tissues, impair membrane functions, affect cellular metabolism, and passivate proteins and enzymes, especially in the organs with fast metabolic processes like the liver and kidney [34,35,36]. Consistent with these studies, we found that repeated d-gal-injection induced an observable decrease in the activities of these key antioxidant enzymes (SOD, CAT, and GSH-Px), and markedly aggravated lipid peroxidation, manifested as the accumulation of MDA, indicating that oxidative damages occurred in mice liver and kidney. Furthermore, the serum levels of ALT, AST, and ALP are widely used to evaluate liver functions, in which ALP plays a very important role in the dephosphorylation of protein enzymes, while ALT and AST are the earliest and most sensitive indicators of liver injury. Meanwhile, UA and CREA are the main indicators of renal function, which will be filtered out through the glomerular fluid into the blood stream in large amounts when renal damage occurs during the aging process [37,38]. Accompanied with oxidative tissue damage of mice liver and kidney, the serum levels of ALT, AST, and ALP, as well as CREA and UA in the model group mice, were significantly higher than those of normal group mice. However, COS, from shrimp shell chitosan by enzymatic hydrolysis, not only exhibited favorable antioxidant properties by enhancing the activities of these antioxidases and decreasing the level of MDA, showing the oppositional effect on d-gal-induced oxidative damages to mice liver and kidney, but also mitigated these exceptional rises of serum ALT, AST, ALP, CREA, and UA levels to an extent lower than the model group, indicating an important role in preserving the normal function of the liver and kidney. Meanwhile, VE also displayed, to a certain extent, an antioxidant effect mainly by intensifying the activities of SOD in mice liver and kidney, as well as reducing the levels of MDA, to implement a degree of protective effects on mice hepatic and renal functions. Additionally, histological examination showed that the levels of inflammatory cell infiltrations and necrosis of hepatocytes, as well as the atrophy of the renal glomerulus were enhanced in the model group, whereas the application of COS and VE ameliorated these hepatic and renal structure damages induced by chronic administration with d-gal to a certain extent, and the histological status in the COS-H group closely resembled that of the normal group. These results of histological assessments corresponded well with the biochemical analysis. 

To sum up, VE, the monomer of which is often used as the positive control for the studies of subacute aging in mice induced by d-gal, is a well-known fat-soluble vitamin with excellent antioxidant activity [37,39,40]. Given that VE showed no prominent promoting effect on these d-gal-produced reduction of immune function indicators of mice, it was reasonable to infer that the certain protective effect of VE on the liver and kidney of d-gal-induced aging mice might be mainly due to its anti-oxidation quality. Relatively, COS, derived from the shells of shrimp and crab shells in the ocean, possesses a variety of biological effects, such as the increasing effect on these d-gal-induced reduction of immune function indicators of mice, and d-gal-caused decreases in the activities of key antioxidant enzymes in mice liver and kidney [12,15,16,17]. To comprehensively conclude our results, it could be regarded that COS possessed hepatoprotective and renoprotective effects on d-gal-induced subacute aging mice to realize its anti-aging activity, which might be associated with the anti-oxidant capability of COS, as well as its good regulatory role on mice immune function to a larger extent.

## 4. Materials and Methods

### 4.1. Materials and Chemicals

COS (from shrimp shell chitosan by enzymatic hydrolysis, average molecular weight ≤1000 Da, degree of deacetylation ≥90%, degree of polymerization of 3–7, water soluble), almost white powder, was purchased from Beijing Zhong Tai He Technology (ZTH Tech, Beijing, China). A certain amount of COS was weighed accurately and dissolved in ultrapure water at room temperature to prepare three different concentrations: 15, 30, and 60 mg/mL. d-gal (purity ≥99%) and vitamin E (VE, purity 95%) were purchased from Sigma-Aldrich (St. Louis, MO, USA). d-gal was dissolved in 0.9% physiological saline for injecting subcutaneously back the neck of mice at the dose of 250 mg/kg body weight, while VE was dissolved in distilled water containing 1% Tween 80 solution. Commercially available kits for IgM, IgG, GSH-Px, SOD, CAT, and protein concentration, as well as MDA, were provided by Nanjin Jiancheng Bioengineering Institute (Nanjing, China). All other chemicals and reagents used in the study were of analytical grade.

### 4.2. Animals

Healthy seven-week-old male KM mice, weighing approximately 30 g, were purchased from the Center of Laboratory Animal Science of Guangdong Province (certificated number: SCXK(Yue)-2013-0002). Mice were housed in the animal laboratory of Guangdong Ocean University according to the guides for the care and use of laboratory animals. Mice were maintained in cages at an ambient temperature of 23 ± 2 °C with 55 ± 10% relative humidity in a 12 h light/dark automatic lighting cycle during the experimental period of eight weeks. They could access to the standard pellet diet and drink water ad libitum throughout the study period.

### 4.3. Preparation of Subacute Aging Mouse Model and COS Treatment

After adaptation for one week, mice were randomly divided into six groups of eight mice each according to Table 3. In order to establish natural aging model mouse, d-gal (250 mg/kg/day) was injected subcutaneously into the back of the neck of mice once a day for 56 consecutive days. While mice in the normal group received equal amounts of physiological saline once daily instead of d-gal. After subcutaneous injection of d-gal in mice for one hour, mice in normal and model groups were treated with distilled water by gavage (20 mL/kg/day); meanwhile, mice in VE group were treated with VE by gavage (50 mg/kg/day) as a positive control according to recent reports [37,39,40]. Mice in COS-L, COS-M, and COS-H groups were treated with the sample solutions (three different doses of COS) per day by gavage, respectively, which were administered daily as the treatment schedule displayed in Table 3.

### 4.4. Daily Observation of Mice

During the experimental period, the body weight of mice was measured by an electronic balance once a week. In addition, the appetite, appearance, mental condition, and behavioral activity of mice in each group were observed and recorded per week, respectively.

### 4.5. Preparation of Mice and Sample Collection 

At the end of the eighth week, 24 h after the last drug administration, the mice were fasted overnight, and their blood samples were taken from the retrobulbar venous plexus. Then, these mice were weighed and then sacrificed by the humane method of cervical dislocation. Subsequently, the spleens, thymus, kidneys, and livers of each mouse were carefully dissected out, washed with cold sterile physiological saline and weighed. The ratio of organ weight to the final body weight was calculated as the organ index. After that, the liver and kidney were stored immediately at −80 °C for the sequent biochemical analysis. There were no casual or obvious signs of toxicity throughout the course of the experiments and all of the mice involved survived. 

### 4.6. Determination of Serum Indices

The serum was separated from the whole blood by centrifugation at 4000 rpm for 15 min at 4 °C and then stored at −80 °C for biological analysis. An automatic biochemistry analyzer (Mindary BS-480, Shenzhen Mindray Bio-Medical Electronics Co., Ltd., Shenzhen, China) was used to measure the levels of ALT, AST, ALP, CREA, UA, IgG, and IgM in the serum of mice according to the assay kit instructions.

### 4.7. Measurement of the Anti-Oxidation Activity in Mice Liver and Kidney

For biochemical analysis, organ tissues of each mouse including liver and kidney were quickly removed to prepare 10% (*w*/*v*) homogenates in ice-cold 0.9% NaCl solution, followed by centrifugation at 3000 rpm for 15 min at 4 °C, and then the supernatant was kept frozen at −70 °C until biochemical analysis. The protein concentrations were measured by bicinchoninic acid (BCA) method using bovine serum albumin as the standard. The activities of SOD, CAT, and GSH-Px, as well as the levels of MDA in liver and kidney, were determined by the assay kits.

### 4.8. Histological Examination of Liver and Kidney 

After sacrifice, organ tissue samples, including liver and kidney, were quickly stripped, fixed in 4% paraformaldehyde for 24 h, dehydrated in ascending grades of alcohol, then embedded in paraffin, and finally sectioned at a thickness of 5 μm. These samples were stained by HE for routine examination of these tissues. 

### 4.9. Statistical Analysis

All quantitative data were presented as means ± SD. Experimental values were analyzed by one-way ANOVA. A value of *p* < 0.05 was considered to be statistically significant. All analyses were performed using Statistical Analysis Software (SPSS 17.0).

## 5. Conclusions

COS, a class of naturally occurring polysaccharides, has a protective effect against d-gal-induced damages of subacute aging in mice mainly by promoting the serum IgG and IgM levels, preventing the atrophy of mice thymus and spleen, as well as increasing the activities of pivotal antioxidant enzymes in mice liver and kidney, revealing that the mechanism might be closely associated with its beneficial modulation of oxidative and immune system. These results may provide supporting pre-clinical evidence for the potential clinical application of COS as a therapeutic product against age-related diseases.

## Figures and Tables

**Table 1:** Effects of COS on the organ indices and immune function in d-gal-treated mice.

Groups	Organ Index (mg/g)		Immune Function	
	Thymus	Spleen	IgG (g/L)	IgM (g/L)
Normal	0.22 ± 0.05	0.58 ± 0.28	0.80 ± 0.03	0.16 ± 0.03
Model	0.14 ± 0.05 ^#^	0.34 ± 0.09 ^#^	0.57 ± 0.04 ^#^	0.07 ± 0.02 ^#^
VE	0.19 ± 0.04	0.43 ± 0.16	0.61 ± 0.05	0.10 ± 0.03
COS-L	0.14 ± 0.04	0.42 ± 0.07	0.70 ± 0.04 *	0.08 ± 0.02
COS-M	0.16 ± 0.06	0.49 ± 0.23 *	0.72 ± 0.07 *	0.11 ± 0.02 *
COS-H	0.19 ± 0.05	0.53 ± 0.14 *	0.77 ± 0.08 *	0.12 ± 0.02 *

Data represent means ± standard deviation (SD) (n = 8). **^#^** *p* < 0.05 compared with the normal group; * *p* < 0.05 compared with the model group.

**Table 2:** Effects of COS on liver and kidney functions in d-gal-treated mice.

Groups	Liver Function			Kidney Function	
	ALP (U/L)	ALP (U/L)	AST (U/L)	CREA (μmol/L)	UA (μmol/L)
Normal	50.08 ± 5.46	20.88 ± 3.17	53.79 ± 4.40	51.46 ± 3.08	125.24 ± 9.52
Model	82.26 ± 3.09 ^#^	28.20 ± 4.39 ^#^	78.51 ± 3.77 ^#^	61.49 ± 5.53 ^#^	162.55 ± 8.93 ^#^
VE	69.26 ± 6.05 *	22.05 ± 4.02 *	68.61 ± 5.24 *	58.36 ± 6.28	142.83 ± 7.76 *
COS-L	68.28 ± 4.57 *	25.59 ± 3.63	65.15 ± 4.21 *	55.51 ± 3.36	160.33 ± 5.55
COS-M	64.07 ± 3.29 *	20.45 ± 2.05 *	60.08 ± 2.26 *	53.14 ± 3.70 *	139.13 ± 11.03 *
COS-H	58.85 ± 4.83 *	19.10 ± 1.52 *	57.01 ± 3.67 *	51.99 ± 1.09 *	130.39 ± 8.65 *

Data represent means ± SD (n = 8). **^#^** *p* < 0.05 compared with the normal group; * *p* < 0.05 compared with the model group.

**Table 3:** Treatment schedule of the study.

Groups	d-Gal (250 mg/kg/day)	COS (mg/kg/day)		
		300	600	1200
Normal	−	−	−	−
Model	+	−	−	−
VE	+	−	−	−
COS-L	+	+	−	−
COS-M	+	−	+	−
COS-H	+	−	−	+

+: With treatment, −: Without treatment.
